# Synaptic cell-adhesion molecule latrophilin-2 is differentially directed to dendritic domains of hippocampal neurons

**DOI:** 10.1016/j.isci.2024.108799

**Published:** 2024-01-16

**Authors:** Thomas R. Murphy, Ryan F. Amidon, Jordan D. Donohue, Libo Li, Garret R. Anderson

**Affiliations:** 1 Department of Molecular, Cell and Systems Biology, University of California, Riverside, Riverside, CA 92521, USA; 2 School of Medicine, Medical College of Wisconsin, Milwaukee, WI 53226, USA; 3 Neuroscience Graduate Program, University of California, Riverside, Riverside, CA 92521, USA

**Keywords:** Molecular neuroscience, Cellular neuroscience

## Abstract

Hippocampal pyramidal cells possess elaborate dendritic arbors with distinct domains that are targeted with input-specific synaptic sites. This synaptic arrangement is facilitated by synaptic cell-adhesion molecules that act as recognition elements to connect presynaptic and postsynaptic neurons. In this study, we investigate the organization of the synaptic recognition molecule latrophilin-2 at the surface of pyramidal neurons classified by spatial positioning and action potential firing patterns. Surveying two hippocampal neurons that highly express latrophilin-2, late-bursting CA1 pyramidal cells and early-bursting subiculum pyramidal cells, we found the molecule to be differentially positioned on their respective dendritic compartments. Investigating this latrophilin-2 positioning at the synaptic level, we found that the molecule is not present within either the pre- or postsynaptic terminal but rather is tightly coupled to synapses at a perisynaptic location. Together these findings indicate that hippocampal latrophilin-2 distribution patterning is cell-type specific, and requires multiple postsynaptic neurons for its synaptic localization.

## Highlights

• Localization patterning of synaptic adhesion molecule latrophilin-2 is cell specific
• Late-bursting CA1 pyramidal cells position latrophilin-2 at distal dendritic tufts
• Early-bursting subiculum pyramidal cells enrich latrophilin-2 at proximal dendrites
• Latrophilin-2 is tightly coupled to synapses at a perisynaptic position

## Graphical abstract

**Figure:**
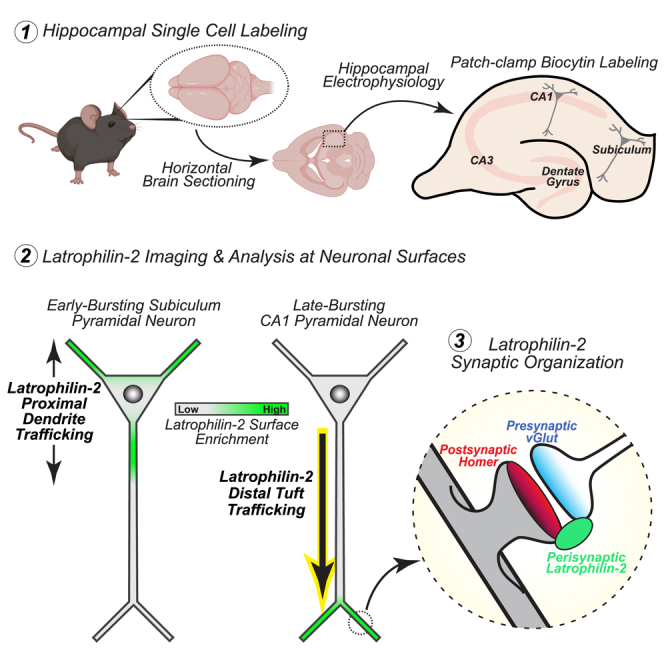

## Introduction

Cortical and hippocampal pyramidal cells perform complex computations made possible by large dendritic trees that receive diverse, yet spatially organized synaptic inputs. Action potential firing patterns of individual neurons are determined by the integration of synaptic inputs across their different cellular compartments, with distinct domains being targeted by specified presynaptic neuron inputs. Hippocampal CA1 pyramidal cells (CA1PCs), for example, receive excitatory glutamatergic synapses from CA3 and the entorhinal cortex targeting proximal and distal dendritic domains, respectively.^1^ Likewise, inhibitory GABAergic synapses arise from various interneuron populations that target CA1PCs in a spatially organized fashion.^2^ This includes basket cells that target CA1PCs at somatic and proximal dendritic regions, bistratified cells that project onto CA1PC dendritic regions in the stratum oriens and radiatum, oriens lacunosum-moleculare (O-LM) cells that target CA1PCs distal tuft dendritic domains, and Chandelier cells that synapse onto CA1PC axons.^3^^,^^4^ Directing this specificity of synaptic organization requires concerted molecular action during multiple stages of circuit development, from neurite outgrowth to synaptogenesis. During the initial stages of synaptogenesis, presynaptic molecules found in incoming axons form extracellular complexes with postsynaptic cell-adhesion molecules that are presented at defined dendritic domains. Participating in this synaptic recognition process is the latrophilin family of adhesion G-protein coupled receptors (Lphn1-3; gene symbols *ADGRL1-3*). In CA1PCs, all latrophilin genes are expressed,^5^^,^^6^^,^^7^ but the translated proteins appear to have distinct cellular distribution patterns. In these neurons, Lphn3 is directed to proximal dendritic domains where it serves to regulate the assembly of CA3 synaptic inputs.^8^ Lphn2, on the other hand, is subject to trafficking mechanisms that enrich it at synaptic sites on distal CA1PC dendritic domains, where it regulates the assembly of entorhinal cortex inputs.^9^ In addition, Lphn2 exhibits regional and cell type-dependent variability in expression across the hippocampus and entorhinal cortex, which is functionally important for topographical axon patterning.^10^^,^^11^^,^^12^ While Lphn2 clearly exhibits cell type-specific and synapse-specific roles in neural circuit development, the nature of subcellular Lphn2 protein trafficking remains poorly understood.

Here, we use single cell labeling and super-resolution confocal imaging to visualize Lphn2 contact points on distinct dendritic compartments of regionally and action potential defined hippocampal neurons that highly express Lphn2, late-bursting CA1 pyramidal cells and early-bursting subiculum pyramidal cells. Surveying the distribution of Lphn2 protein on the surface of these neurons, we find distinct localization patterning between these structurally similar cells. Whereas CA1PCs show strong Lphn2 enrichment in distal dendritic domains, subiculum cells on the other hand enrich at proximal primary dendritic localizations. Using immunofluorescence to label synaptic markers, we observed that the Lphn2 signals in CA1PC distal dendrites occupy a distinct position in the synapse which does not directly overlap with pre- or postsynaptic markers and is approximately equidistant from both. Lphn2 was also detected in close proximity to dendrites of nearby stratum lacunosum-moleculare (SLM) interneurons, with branch-order distribution, even though these cells do not express Lphn2 themselves. These data suggest that Lphn2 expressed in CA1PCs may exhibit cross-talk with adjacent dendrites, and can participate in synapses on both CA1PCs and on non-Lphn2-expressing neurons. In total, our findings suggest a complex role for Lphn2 in the hippocampus, mediated both by perisynaptic cell-cell interactions and by neuron-specific and dendritic domain-specific organization.

## Results

### Hippocampal Lphn2 expression is spatially organized in the CA1 and subiculum

**Figure 1:**
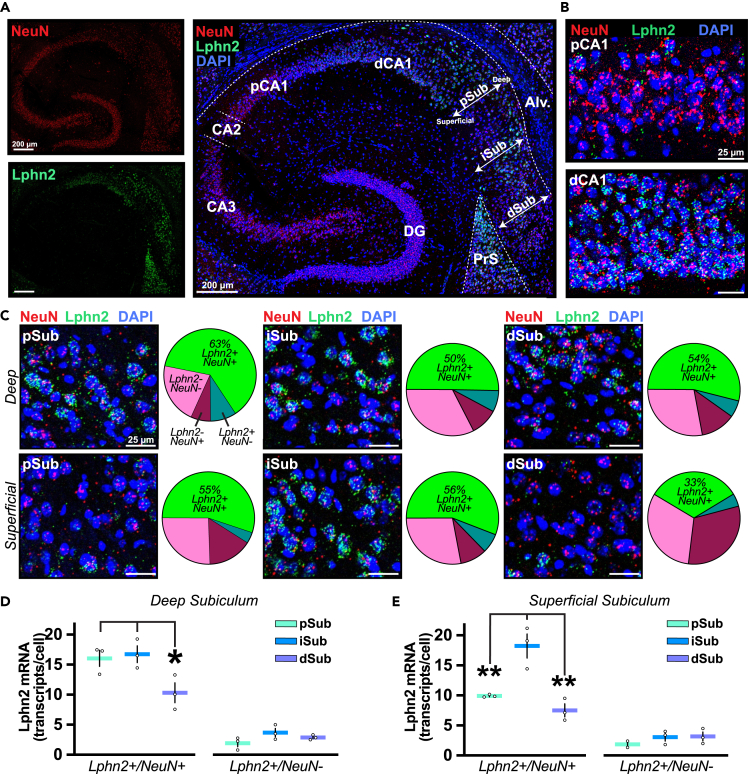
Hippocampal Lphn2 mRNA expression is spatially organized in the CA1 and subiculum (A) Single molecule fluorescent *in situ* hybridization (smFISH) with mRNA probes for NeuN (a neuronal marker) and Lphn2 in a horizontal hippocampal slice. The indicated regions include the presubiculum (PrS), dentate gyrus (DG), CA3, proximal CA1 (pCA1), distal CA1 (dCA1), and proximal/intermediate/distal subiculum (pSub, iSub, dSub). White arrows denote the superficial-to-deep axis along which subiculum regions were subdivided, with deep regions being most proximal to the alveus. (B) Lphn2 expression in pCA1 versus dCA1 of a representative hippocampal section. (C) Representative images and cell type classification percentages for subiculum regions pSub (*left*), iSub (*middle*), and dSub (*right*), subdivided into deep (top row) and superficial (bottom row) subregions. Cells are classified into one of four categories based on the expression or absence of NeuN and Lphn2. (D and E) Summary graphs of Lphn2 expression levels within Lphn2 expressing neurons (Lphn2+/NeuN+) and nonneuronal cells (Lphn2+/NeuN-) of pSub/iSub/dSub areas, subdivided into deep (D) and superficial subregions (E). Data shown are means +/−SEMs (n = 3 mice). Statistical analysis was performed by one-way ANOVA with Tukey’s post hoc test for multiple comparisons (∗p < 0.05, ∗∗p < 0.01).

To further our understanding of Lphn2 organization in the hippocampus, we first sought to examine which neurons genetically express Lphn2. Using single molecule fluorescent *in situ* hybridization (smFISH), we surveyed intermediate hippocampal Lphn2 mRNA expression in horizontal (∼2.6 mm ventral from bregma) mouse brain sections (Figure 1A). Consistent with previous reports, we observed that Lphn2 expression was organized topographically across the hippocampal CA1 pyramidal cell layer.^9^^,^^10^^,^^11^ Lphn2 expression in the CA1 pyramidal layer exhibited a proximal-distal expression gradient, with little to no expression in CA1PCs more proximal to CA3 (pCA1PCs; Figure 1B, top) and enrichment in distal CA1PCs (dCA1PCs; Figure 1B, bottom). In the subiculum distal to dCA1, Lphn2 expression was also found to be enriched. However, unlike in CA1, Lphn2 expression in the subiculum did not exhibit a clear proximal-distal expression gradient and instead appeared to be subdivided into multiple specific subregions. To quantify this expression patterning, we first divided the subiculum into three regions along the proximal-distal axis from CA1 to presubiculum (PrS). These regions were defined as proximal (pSub), intermediate (iSub), and distal (dSub) subiculum, based on their locations relative to CA1 (Figure 1A). To account for the reported subiculum pyramidal cell heterogeneity along the superficial-to-deep axis of the subiculum,^13^ we further subdivided each of the subiculum regions above into “deep” and “superficial” subregions, with the deep subregion being most proximal to the alveus (Figure 1A). Using multiplex smFISH for Lphn2 and the neuronal nuclei marker NeuN (Rbfox3), we classified cells in each of these six subiculum subregions as neurons (NeuN+) or nonneuronal cells (NeuN-) which were either positive (Lphn2+) or negative (Lphn2-) for Lphn2 expression (Figure 1C). This cell classification strategy revealed the vast majority of Lphn2+ cells to be neurons across all subregions of the subiculum. The highest density of Lphn2+ neurons was found in the deep pSub subregion (63%), while the lowest density was found in the superficial dSub (33%). All other subregions exhibited a relatively consistent density of Lphn2+ neurons (50–56%).

Next, we analyzed Lphn2 expression levels within the Lphn2+ cell population for each subregion (Figures 1D and 1E). Lphn2 expression levels were significantly higher in neurons (13.2 ± 0.8 transcripts/cell; mean ± SEM, n = 3 animals) than nonneuronal cells (2.8 ± 0.2 transcripts/cell) across all subregions (Figures 1D and 1E). In the deep subregions, dSub expression levels were lower than those of pSub and iSub (Figure 1D). In superficial subregions, we found significant Lphn2 enrichment in iSub compared to the flanking pSub and dSub regions (Figure 1E). These findings indicate that Lphn2 expression is selectively and topographically organized in both CA1 and subiculum neuron populations, with dCA1 and iSub consistently expressing the highest levels of Lphn2.

### Lphn2 protein is differentially organized on dCA1 and iSub pyramidal cells

**Figure 2:**
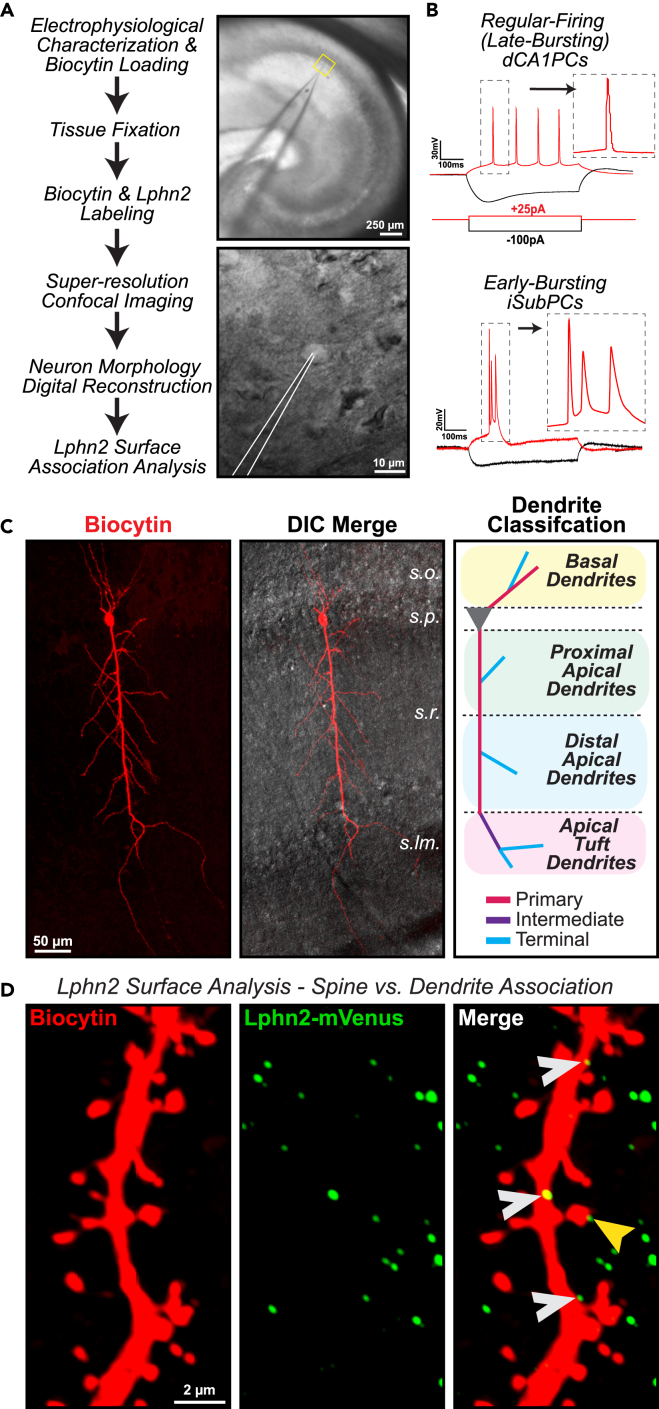
Method for cellular identification and Lphn2 analysis at neuronal surfaces (A) *Left,* General workflow for analyzing Lphn2 surface contact points on isolated neurons in acute hippocampal slices from Lphn2^mVenus^ mice. *Right,* Example DIC images captured during patch clamp recording and biocytin loading of a dCA1 neuron. (B) Representative electrophysiological recordings of action potential firing patterns from a regular-firing (late-bursting) dCA1PC (*top*) and an early burst-firing iSubPC (*bottom*) when held above the firing threshold. (C) *Left*, Fluorescent image of an immunostained biocytin-loaded dCA1 neuron. *Middle,* DIC image overlay with regions indicated (s.o., stratum oriens; s.p., stratum pyramidale; s.r., stratum radiatum; s.lm., stratum lacunosum-moleculare). *Right*, Illustration of general pyramidal neuron functional dendritic domain classifications (Basal, Proximal Apical, Distal Apical, and Apical Tuft) and branch subdivisions (Primary, Intermediate, and Terminal). (D) Sample images of a biocytin-loaded dendrite immunostained for Lphn2-mVenus. Lphn2 surface contact points along dendrite domains were identified using 3-dimensional image reconstruction software and classified into dendrite shaft-associated (*white arrow*) or spine-associated contacts (*yellow arrow*).

With similar Lphn2 mRNA expression that is observed between dCA1 and iSub regions (Figure 1), we next set out to survey the fate of Lphn2 protein from these neurons. The subiculum consists of loosely organized pyramidal neuron and interneuron populations, with pyramidal cells identifiable by large cell bodies and prominent apical dendrites.^14^ Given the morphological comparability between dCA1PCs and iSub pyramidal cells (iSubPCs), we asked whether Lphn2 is distributed across the pyramidal neuron architecture in a similar manner for both cell types. To investigate this, we began by performing single-cell labeling experiments for both dCA1PCs and iSubPCs to analyze Lphn2 protein localization at the surface of these neurons (Figure 2). To visualize Lphn2 protein, we utilized a genetic knock-in mouse model (Lphn2^mVenus^) that forces the expression of a defined isoform of Lphn2 with a mVenus fluorescent tag integrated into the intracellular C-terminal region.^9^ To fully characterize pyramidal neurons of the CA1 and subiculum, during our experiments we sought to differentiate them not only by their spatial positioning, but also by the electrophysiological properties that have been implicated to represent distinct neural circuit pathways.^15^ The CA1 and subiculum consist of two distinct pyramidal cell-types that are identifiable by their action potential firing patterning: (1) pyramidal cells that fire single action potentials with initial depolarization, but exhibit burst-firing patterns with repeated stimulation classified as Regular Firing – Late Bursting; and (2) pyramidal cells that fire a burst of >2 action potentials with initial depolarization, classified as Early Bursting.^16^^,^^17^ To be able to distinguish between these two pyramidal neuron types, we utilized whole-cell patch clamp electrophysiology in acute hippocampal slices from Lphn2^mVenus^ animals to isolate and label individual neurons with biocytin infusion through the patch pipette (Figure 2A). Beginning with the dCA1, we surveyed the firing patterns of these cells with stepwise current injections and found that the vast majority (7 out of 7 recorded) exhibited single action potential firing patterns upon reaching minimal current injection threshold and classified accordingly as Regular Firing – Late-Bursting cells (Figure 2B). In contrast, the majority (7 out of 10 recorded) of iSubPCs in our experiments fired bursts of >2 action potentials followed by rapid adaptation and were classified as Early-Bursting pyramidal neurons (Figure 2B). For our subsequent analysis, we focused on comparing Regular Firing – Late Bursting dCA1PCs with Early-Bursting iSubPCs for Lphn2 visualization. After electrophysiological characterization, the tissue was fixed and labeled for biocytin and Lphn2. We then systematically imaged distinct dendritic compartments of biocytin-labeled neurons using 3-dimensional super-resolution confocal microscopy.^18^

Diverse and compartmentalized pyramidal dendritic branch-specific properties allow for circuit-specific responses to spatiotemporal patterns of synaptic input.^19^^,^^20^^,^^21^ Accordingly, we performed a detailed analysis of Lphn2 sites defined by branch specified positions. Pyramidal cell dendritic trees can be broadly divided into basal and apical dendrite compartments, and subdivided into primary, intermediate, and terminal branches.^2^ Dendritic branches were defined as primary if they were directly connected to the soma, terminal if they contained no further branch points, and intermediate if they did not fall into the primary or terminal categories (Figure 2C). Since apical dendrites are extremely long and can span multiple functionally distinct regions, we further subdivided the apical dendrite compartment into three distinct segments: proximal apical, distal apical, and tuft apical dendrites. Using 3-dimensional image analysis software, we generated digital reconstructions of the biocytin and Lphn2 signals in our acquired images and analyzed Lphn2 signals associated specifically at the surface (<0.5 μm) of the labeled neuron’s dendrite or dendritic spines (Figure 2D). In doing so, we are visualizing Lphn2 sites that are likely to be positioned at possible synapses onto the identified neuron, but not limited to solely postsynaptic localization.

**Figure 3:**
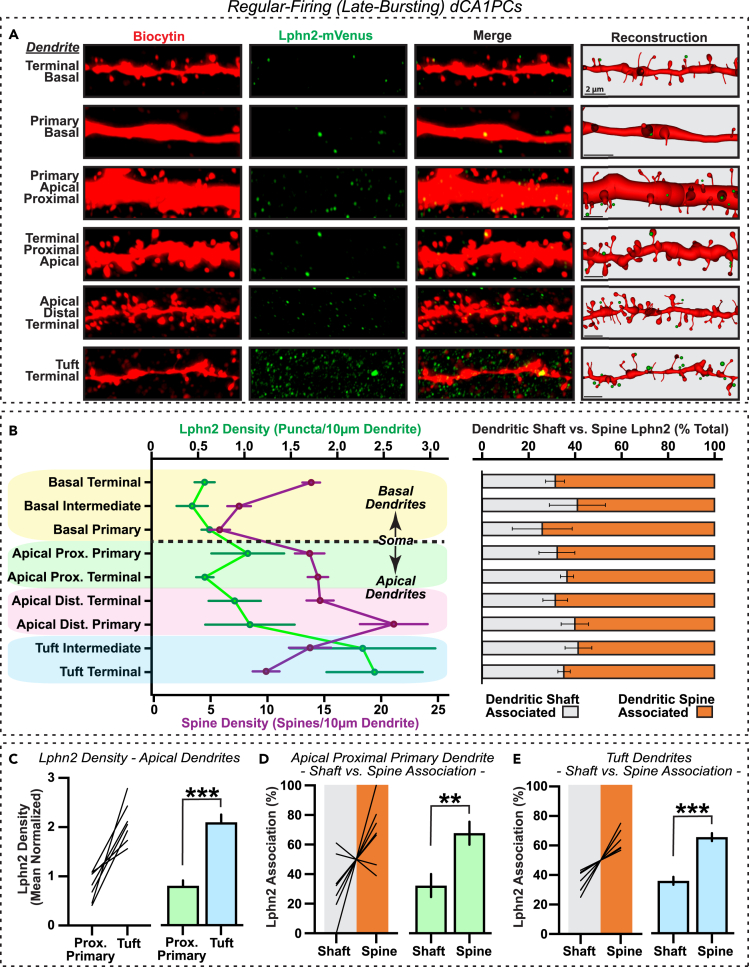
Lphn2 is localized to distal tuft dendrites of late-bursting CA1 pyramidal neurons (A) Representative images of biocytin and Lphn2-mVenus immunofluorescence at various dendritic subdomains of a sample dCA1PC. Three-dimensional reconstructions of biocytin and Lphn2 signals are displayed in the right column. (B) *Left,* Average Lphn2 puncta (green) and spine densities (purple) quantified for each domain/subdomain category across all imaged late-bursting dCA1PCs. *Right,* Dendrite-associated Lphn2 is classified into dendrite shaft-associated versus spine-associated Lphn2 contacts for each dendrite category. (C) Lphn2 puncta densities for proximal apical primary and apical tuft dendrites (normalized by total Lphn2 density across all dendritic domains), displayed as individual neuron plots (*left*) and summary graphs (*right*). (D and E) Individual neuron plots (*left*) and summary graphs (*right*) showing percentages of Lphn2 puncta associated with dendrite shafts versus spines in proximal primary dendrites (D) and tuft dendrites (E). Data shown are means ± SEMs (n = 7 neurons). Statistical analysis was performed by Mann-Whitney test (∗∗p < 0.01; ∗∗∗p < 0.001).

**Figure 4:**
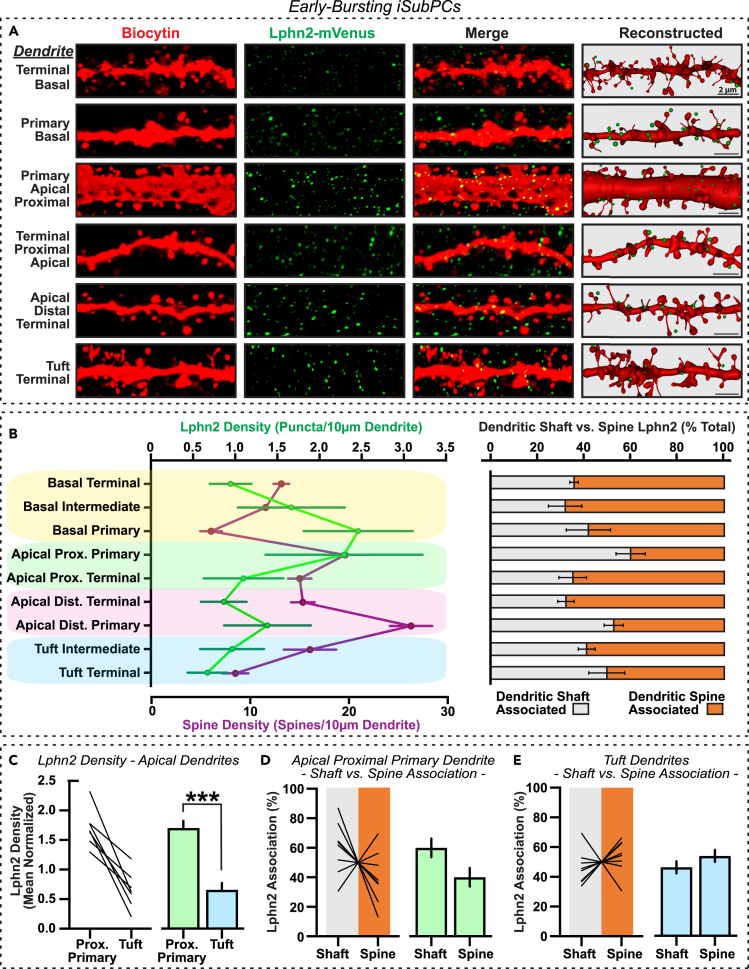
Lphn2 is localized to proximal primary dendrites in early-bursting iSubPCs (A) Representative images of biocytin-labeled dendrites and Lphn2 puncta, and their digital reconstructions, for various dendrite subdomains of a sample early-bursting iSubPC. (B) *Left,* Average Lphn2 puncta (green) and spine densities (purple) quantified across domain/subdomain classifications for all imaged early-bursting iSubPCs. *Right,* Percentages of dendrite-associated Lphn2 puncta classified as dendrite shaft-associated versus spine-associated for all dendrite categories. (C) Lphn2 puncta densities in proximal apical primary and tuft dendrites (normalized by total Lphn2 density across all dendritic domains), displayed as individual neuron plots (*left*) and averages (*right*). (D and E) Individual neuron plots (*left*) and summary graphs (*right*) of Lphn2 associated with dendrite shafts versus spines in proximal primary dendrites (D) and tuft dendrites (E), as a percentage of total puncta associated with each dendrite type. Data are displayed as means ± SEMs (n = 8 neurons). Statistical analysis was performed by Mann-Whitney test (∗∗∗p < 0.001).

Systematically analyzing these parameters, we proceeded to examine the distribution of Lphn2 contact points across dendritic compartments of late-bursting dCA1PCs (Figure 3) and early-bursting iSubPCs (Figure 4). Consistent with the enriched Lphn2 protein expression observed in the stratum lacunosum moleculare region,^8^^,^^9^ we observed a significant increase in dendrite-associated Lphn2 in apical tuft compartments of late-bursting dCA1PCs compared to other dendritic locations (Figures 3A, 3B, and S1A). In all dendritic compartments, Lphn2 was found to preferentially associate with spines (∼65%) rather than dendritic shaft locations (∼35%) (Figure 3B). To examine this distribution pattern in greater detail, we compared Lphn2 density and localization between two representative apical dendrite compartments, proximal primary and tuft, within single neurons. Despite the significantly lower Lphn2 density in the proximal dendritic compartment (Figure 3C), Lphn2 was localized preferentially at spine locations in both proximal (∼68%; Figure 3D) and tuft dendrite compartments (∼62%; Figure 3E). Taken together with the observed Lphn2 mRNA expression pattern in CA1 (Figure 1), these findings suggest that Lphn2 protein in CA1 is expressed predominantly by dCA1PCs and undergoes robust intracellular trafficking primarily to spines in the dendritic tuft.

In a similar fashion, we surveyed the Lphn2 distribution on iSubPCs (Figure 4). Focusing on early-bursting cells, the predominant cell type in the subiculum (Figure 2B), we measured Lphn2 density and sub-dendritic localization in each of the previously defined pyramidal cell dendritic compartments (Figure 2C). In contrast to late-bursting dCA1PCs, Lphn2 enrichment in early-bursting iSubPCs was found primarily in the primary dendrites of basal and proximal apical compartments (Figures 4A and 4B). Additionally by surveying across the dendritic compartments in early-bursting iSubPCs, Lphn2 spine vs. shaft localization appears to exhibit greater branch specific variation and patterning that is distinct from dCA1PCs. Within-cell comparisons between apical proximal primary and tuft dendrites revealed Lphn2 enrichment to be preferentially localized to proximal primary dendrites in early-bursting iSubPCs (Figures 4C, S1B, and S1C) as opposed to the tuft dendritic enrichment observed in late-bursting dCA1PCs (Figures 3C, S1A, and S1C). Further, early-bursting iSubPCs (Figures 4D and 4E) appear to lack the spine localization partiality that is observed in late-bursting dCA1PCs (Figures 3D and 3E). These findings suggest that despite the comparable expression levels of Lphn2 in both iSubPCs and dCA1PCs (Figure 1), these neurons possess distinct subcellular trafficking mechanisms for the protein.

### Hippocampal Lphn2 is positioned at synaptic sites at a perisynaptic position

**Figure 5:**
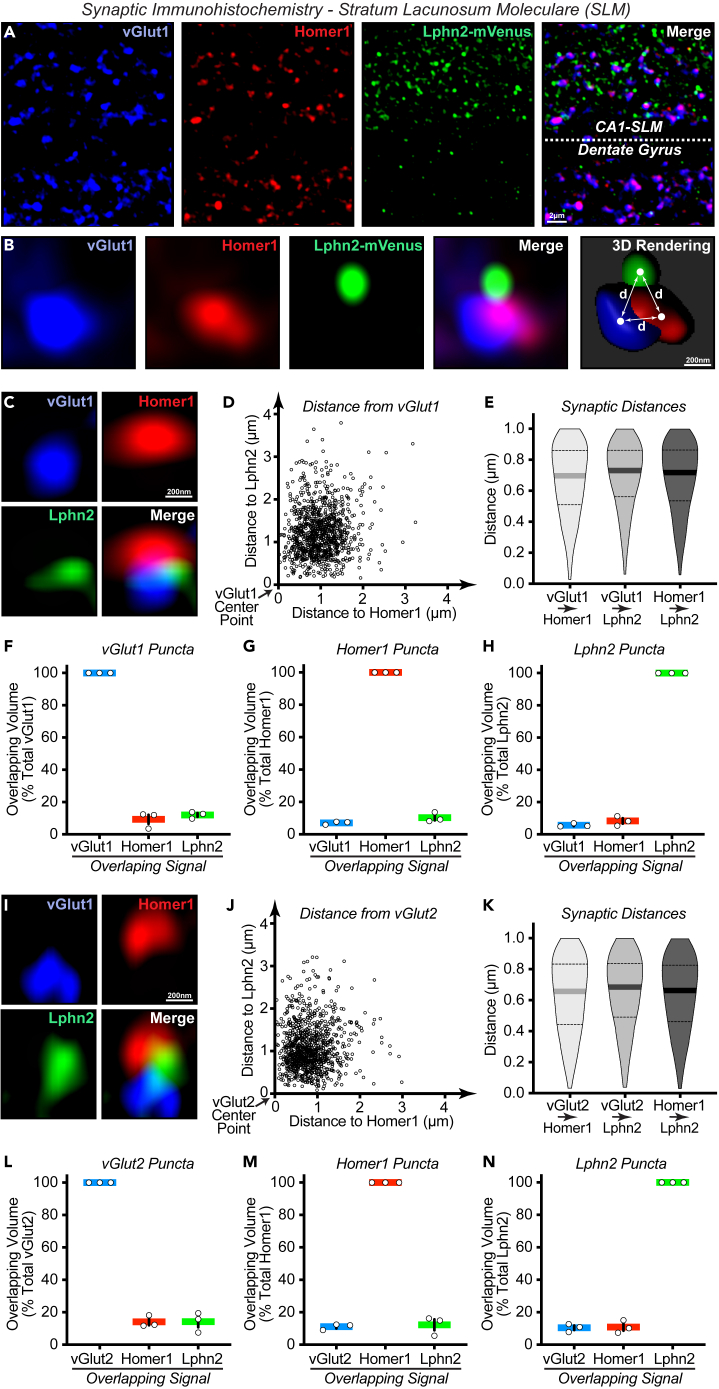
Hippocampal Lphn2 is localized to synapses at perisynaptic positions (A) Immunofluorescence staining of the CA1-SLM bordering the dentate gyrus in Lphn2^mVenus^ mice using antibodies against vGlut1, Homer1, and GFP (Lphn2) to assess Lphn2 association with excitatory synaptic markers. (B) Zoomed image of an individual synaptic site with all three labels present (vGlut1, Homer1, Lphn2) and 3D reconstruction. Distances (d) between synaptic markers were calculated based on three-dimensional center point values from punctate reconstructions of synaptic markers. (C) Representative image of Lphn2, vGlut1, and Homer1 triple labeling in CA1-SLM. (D) Minimum distances calculated from each vGlut1 puncta to the nearest Homer1 (x axis) and Lphn2 (y axis), plotted for a randomized subset of vGlut1 puncta (n = 1000; 3 animals). (E) Violin plots showing distributions of minimum distances between each pair of markers. Analysis was limited to a randomized subset of markers within 1 μm of one another (n = 300; 3 animals). Mean distance is shown as solid thick line, and dotted lines indicate 25% and 75% quartiles. (F–H) Overlapping volume of vGlut1 (F), Homer1 (G), and Lphn2 (H) with the indicated overlapping signal. Data are displayed as means +/- SEMs (n = 3 animals). (I–N) Similar to (C–H), but for vGlut2/Homer1/Lphn2 triple labeling experiments.

To investigate hippocampal Lphn2 organization in finer detail, we next set out to characterize Lphn2 localization within synaptic sites. Previous work has demonstrated that Lphn2 plays a critical role in hippocampal excitatory synaptic transmission, with its absence leading to a loss in synaptic strength at entorhinal cortex glutamatergic inputs onto CA1PCs positioned within the SLM.^8^^,^^9^ Accordingly, we began by visualizing Lphn2 in relation to excitatory synaptic markers within the SLM. To do so, we performed triple labeling immunofluorescence to detect Lphn2 alongside presynaptic terminal marker vesicular glutamate transport protein (vGlut1 or vGlut2), and postsynaptic density marker Homer-1. Subsequently utilizing superresolution confocal microscopy and 3D image analysis, we obtained high-accuracy representations of Lphn2 signal in relation to these pre- and postsynaptic markers in space (Figures 5A and 5B). During these experiments, we observed a consistent patterning of Lphn2 localization. In both vGlut1-containing and vGlut2-containing synapses, Lphn2 appears to be localized in close proximity to the synapse, but at a position distinct from both the presynaptic terminal and postsynaptic density. To determine the spatial relationship between Lphn2 with pre- and postsynaptic excitatory markers, using 3D image analysis we digitally reconstructed and calculated the distance to the other two markers (Figure 5B). For vGlut1 containing synapses, we find vGlut1 to be non-overlapping with both Homer-1 and Lphn2 marked sites (Figures 5C–5H). Analyzing synapses where all three markers (vGlut, Homer-1, and Lphn2) were localized within a realistic synaptic distance of each other (<1 μm), we found that Lphn2 was located at equal distances from vGlut1 (693 ± 14 nm; mean ± SEM, n = 3 animals) and Homer-1 (681 ± 5 nm) (Figure 5E). This distance was comparable to the distance between synaptic markers vGlut1 and Homer-1 (666 ± 18 nm) (Figure 5E). While positioned in close proximity to one another, each of the three markers (vGlut1, Homer1, and Lphn2) exhibits largely independent signal with only slight overlap (∼6–12%) (Figures 5F–5H). For vGlut2/Homer1 synapses, a similar pattern emerges (Figures 5I–5N). Lphn2 was also found to be equidistant between vGlut2 (646 ± 17 nm; mean ± SEM, n = 3 animals) and Homer-1 (636 ± 10 nm), comparable to the coupling distance between vGlut2 and Homer-1 (625 ± 11 nm) (Figure 5H). Similarly, vGlut2 (Figure 5L), Homer 1 (Figure 5M), and Lphn2 (Figure 5N) exhibited largely independent signal, and sharing slight overlap (∼10–14%) with the other two markers. Thus, while Lphn2 shows a strong association with both vGlut1-containing and vGlut2-containing excitatory synapses within the CA1-SLM, it appears to localize to a perisynaptic position equidistant from the presynaptic and postsynaptic sites. These results indicate that Lphn2 is not localized as a cell-adhesion molecule acting directly to bridge the pre- and postsynaptic neuron at the synaptic interface, but rather arises indirectly from an adjacent cell to form synaptic adhesion complexes in a perisynaptic fashion.

**Figure 6:**
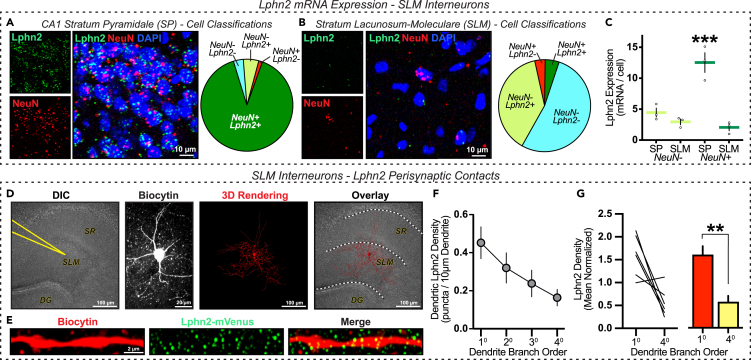
SLM interneurons exhibit Lphn2 contact points that are organized in dendritic branch order (A–C) *SLM interneurons are Lphn2 mRNA deficient.* Representative images (*left*) and cell classifications (*right*) from smFISH experiments with NeuN and Lphn2 mRNA probes in the (A) CA1 stratum pyramidale (SP) and (B) stratum lacunosum-moleculare (SLM). (C) Summary graphs of Lphn2 expression levels in Lphn2+ nonneuronal (NeuN-) and neuronal (NeuN+) cell populations in the SP and SLM. Data shown are means +/−SEMs (n = 3 mice). Statistical analysis was performed by one-way ANOVA with Tukey’s post hoc test for multiple comparisons (∗∗∗p < 0.001). (D–G) *Lphn2 protein exhibits close contacts with SLM interneurons*. (D) Representative DIC image showing the approximate location of a sample SLM interneuron (*left*) targeted for electrophysiological recording and biocytin loading (*middle left*); 3D digital reconstruction (*middle right*); Overlay of interneuron 3D rendering onto DIC image (*right*). (E) Representative biocytin and Lphn2 labeling along an SLM interneuron dendrite. (F) Lphn2 contact point density quantifications for primary (1°), secondary (2°), tertiary (3°), and quaternary (4°) dendritic branches. (G) Single-cell (*left*) and summary graphs (*right*) of Lphn2 densities for 1° and 4° branch orders, normalized by total Lphn2 density across all dendrites. The data shown are the means +/− SEMs (n = 6 neurons). Statistical analysis was performed by Mann-Whitney test (∗∗p < 0.01).

We postulated that this Lphn2 perisynaptic cross-talk principle would exist not only between two Lphn2 expressing neurons, but also between Lphn2-expressing neurons and non-Lphn2-expressing neurons. To explore this possibility, we next sought to examine the CA1-SLM region in further detail. The CA1-SLM is composed of dendritic elements mainly from two sources: distal tuft dendrites from CA1 pyramidal neurons and local dendrites from SLM interneurons.^22^^,^^23^ Using smFISH to survey Lphn2 mRNA expression in these cell types, we again found that the distal CA1 pyramidal cell layer was abundant in Lphn2-expressing neurons (∼90%; Figure 6A). The interneurons positioned in the SLM on the other hand, exhibited very low positivity for Lphn2 detection (∼5%; Figure 6B). Even in those SLM interneurons that did express Lphn2 transcripts, they were ∼7X more abundant in CA1 pyramidal cells than SLM interneurons (Figure 6C). Lphn2 counts were very low in SLM interneurons (2.1 ± 0.5 transcripts/cell; mean ± SEM, n = 3 animals) and comparable to Lphn2 counts observed in non-neuronal SLM cells (2.9 ± 0.5 transcripts/cell), further indicating the lack of functional Lphn2 expression in the region. Utilizing patch clamp biocytin labeling and Lphn2 detection once again, we captured superresolution images of SLM interneuron dendrites and performed 3D image analysis to evaluate Lphn2 association with SLM interneuron dendrites (Figures 6D and 6E). Similar to pyramidal cells, we divided our analyses by branch order (primary, secondary, tertiary, and quaternary) to ensure functional differences in Lphn2 association would be detected. Through these analyses, we found that Lphn2 association followed a consistent pattern across all SLM interneurons surveyed. Lphn2 puncta were most strongly associated with primary dendrites, with a steady decline with increasing branch order (Figures 6F, 6G, and S2). Despite lacking functional Lphn2 expression, SLM interneurons associate with Lphn2 on its dendritic surface and are organized into distinguishable dendritic compartments.

## Discussion

**Figure 7:**
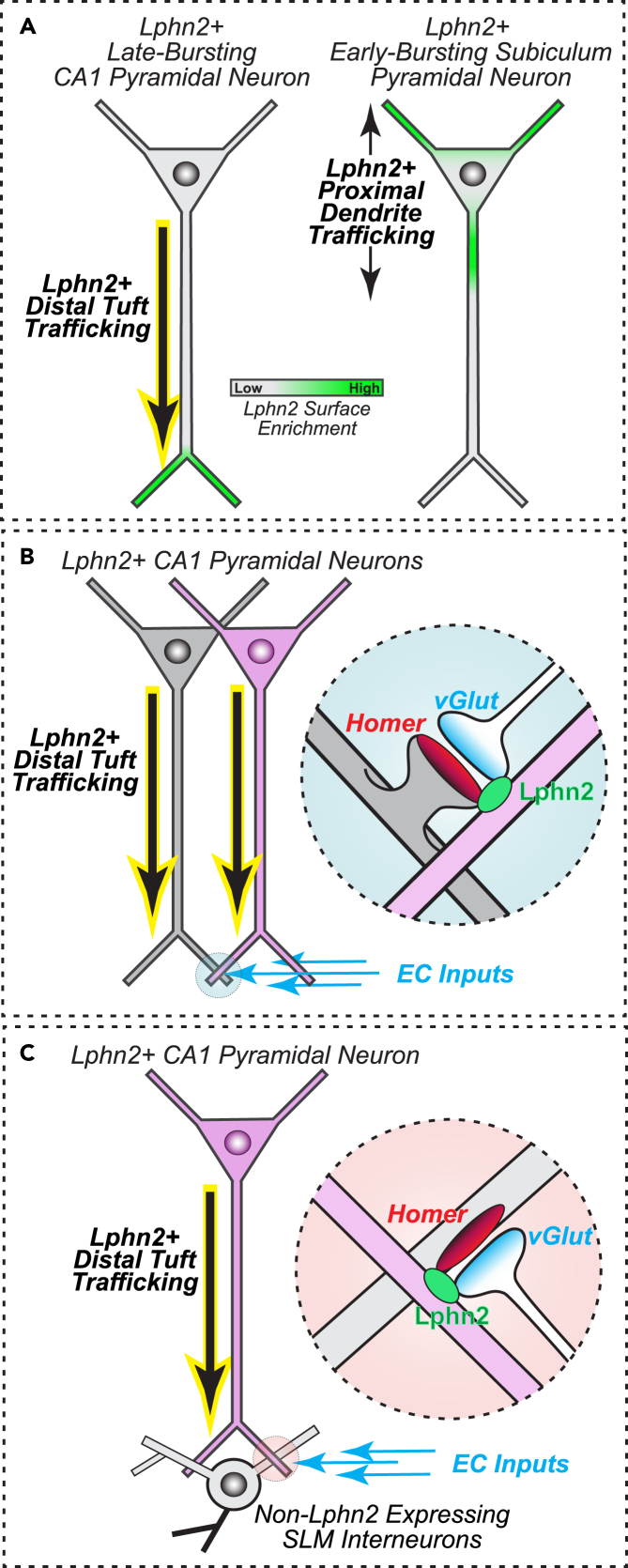
Model of Lphn2 localization patterning in hippocampal neurons (A) Differential Lphn2 positioning between hippocampal late-bursting dCA1 and early-bursting iSub pyramidal neurons. Lphn2 in late-bursting dCA1PCs is uniquely directed to its distal tufts (*left*), while early-bursting iSubPCs localize Lphn2 preferentially to its proximal primary dendrites (*right*). (B and C) Proposed models for how dCA1PC-expressed perisynaptic Lphn2 may interact indirectly with neighboring synaptic sites, such as entorhinal cortex (EC) excitatory inputs onto adjacent dCA1PCs (B) or Lphn2 non-expressing SLM interneurons (C).

By analyzing the spatial distribution of Lphn2 on specific dendritic branches within hippocampal neurons, this study has revealed two key features of the organization of this synaptic cell-adhesion molecule. First, we observed significant differences in Lphn2 localization patterns on dendritic branches between pyramidal cells in the hippocampal CA1 and subiculum. Late-bursting CA1PCs exhibit strong intracellular trafficking of Lphn2 to distal tuft dendrites, while early-bursting iSubPCs localize Lphn2 preferentially to their proximal primary dendrites (Figure 7A). This suggests that Lphn2 may have different functional roles in synaptic development and maintenance for each neuron type. Second, in analyzing Lphn2 at synaptic sites in the CA1-SLM region, we found Lphn2 was localized equidistantly from both pre- and postsynaptic compartments of glutamatergic synapses regardless of type (i.e., vGlut1 vs. vGlut2). These findings indicate that Lphn2 functions as a perisynaptic cell adhesion molecule at SLM glutamatergic synapses, acting indirectly at these sites rather than directly forming cell-adhesion complexes at the presynaptic to postsynaptic interface.

Given its perisynaptic localization, Lphn2 does not appear to fit a simple pre- to postsynaptic cell adhesion model. Rather, Lphn2 appears to participate in a more complex model that involves neighboring cellular and noncellular interactions at synapses to support their development and function. These perisynaptic interactions include protein-dense extracellular matrix and nonneuronal cellular interactions with astrocytic glia.^24^^,^^25^ Furthermore, electron microscopy imaging of synapses indicates that synaptic sites are surrounded by not only astrocyte contacts but also neuronal dendritic and axonal elements.^26^ The complexity of Lphn2 synaptic function is perhaps best illustrated by our findings for dCA1PCs. In these neurons, the Lphn2 protein is translated but is subject to trafficking mechanisms that position it at its distal dendrites. There it positions at synaptic sites, but in a cross-neuron manner. In this model, Lphn2-expressing dCA1PCs crosstalk with adjacent Lphn2-expressing dCA1PCs (Figure 7B), as well as Lphn2-expressing dCA1PCs to Lphn2-nonexpressing CA1-SLM neurons (Figure 7C). While this is more complicated than a traditional model of pre- to postsynaptic adhesion to bridge synaptic sites, it brings in additional cell types to participate in the function of synaptic adhesion molecules. This permits for complex cellular adhesion, while also reducing extracellular protein in the synaptic cleft that would interfere with efficient synaptic communication.

Accumulating lines of evidence suggest that latrophilin molecules participate in a variety of developmental processes that involve attraction and repulsion cellular signaling including cell migration, axon guidance, and synaptogenesis.^8^^,^^9^^,^^10^^,^^27^ In the subiculum, Lphn2 deletion or overexpression alters axonal topographical target patterning that exists between the CA1 and subiculum.^10^^,^^28^ By functioning as G-protein coupled receptors,^12^^,^^29^ latrophilin functions are magnified by signaling amplification. However, by all accounts, latrophilin effects are cell-type specific and compartmentalized to distinct neuronal regions. Lphn2 deletion in CA1 pyramidal neurons, for example, leads to a selective loss of spine structures and synapses from the entorhinal cortex that form in the SLM region.^9^ Lphn3 deletion in these neurons, on the other hand, leads to selective loss of stratum radiatum synapses arising from the CA3 neurons.^8^ The results from this study suggest that trafficking of the Lphn2 protein to defined dendritic locations is context-dependent. CA1 pyramidal neuron trafficking is unique in driving its expression to its distal tufts. While the molecular determinants responsible for this localization pattern are currently not fully understood, it likely requires both intracellular and extracellular macromolecular interacting partners. Consistent with this idea, there appears to be a developmental timing delay for Lphn2 trafficking to the SLM, which is not fully sequestered until after day 7 of postnatal brain development.^11^ This timing delay, coinciding with the timing of the entorhinal cortex to SLM circuit development,^30^ suggests that pre- to post-synaptic extracellular interactions are required for Lphn2 sequestration at distal tuft dendritic branch locations.

To further our understanding of latrophilin roles in brain development, future studies will be required to carefully dissect extracellular interactions serving as stable complexes for synaptogenesis from those that serve as repulsive signals. Latrophilins form large extracellular macromolecular complexes with a growing number of cell-adhesion protein binding partners that include teneurins (Ten1-4),^31^^,^^32^ fibronectin leucine-rich transmembrane proteins (FLRT1-3),^33^ Uncoordinated-5 (Unc5a-d),^34^^,^^35^ and neurexins.^36^ While the teneurin-FLRT-latrophilin complex can exist as a ternary complex,^27^^,^^37^ alternative complexes such as latrophilin-FLRT-Unc5^34^^,^^35^^,^^38^ are likely competitive in nature. Further complicating macromolecular complex dynamics and competition is their functionality. Balancing attraction/repulsion function, FLRT, Unc5, and Lphn interactions have been found to exhibit repulsive effects on migrating cortical neurons that occur during embryonic and early postnatal development.^27^^,^^34^^,^^38^^,^^39^^,^^40^ Likewise, Ten3 and Lphn2 interactions have been implicated as repulsive cues during axon guidance in hippocampal circuits.^10^^,^^28^^,^^41^ In contrast, at later stages of circuit development, FLRT3, Lphn2/3, and Ten2 have been proposed to work in concert with one another to guide synaptic recognition and formation onto hippocampal CA1 neurons.^8^ Together, these lines of evidence suggest that certain extracellular latrophilin complexes may be transient and serve as repulsive cues during development, but other interactions may serve as long-lasting cues and mediate synapse formation and maintenance. To realize the full scope of these molecular interactions and their role in controlling neural circuits will require an in-depth analysis of which cell types express which genetic components, the developmental points at which they are expressed, and their patterns of intracellular trafficking. This information will improve our understanding of these complexes and the multifunctional roles they play during cell migration, axon guidance, synaptic targeting, and synaptogenesis.

### Limitations of the study

Limitations of the study include the following. (1) Use of Lphn2^mVenus^ mouse model, a genetic knock-in mouse model that is not necessarily a reflection of endogenously expressed Lphn2 protein. This mouse model forces the expression of a single splice isoform under the control of the Lphn2 promoter, with the mVenus tag positioned in the intracellular C-terminal region of the molecule. Adhesion-GPCRs possess an autoproteolytic site at the extracellular region adjacent to the plasma membrane, therefore our results described here may or may not vary if the mVenus tag was positioned in the extracellular N-terminal region. (2) With the imaging methods used in this study and a fixed distance threshold (<0.5 μm from the neuronal surface), assessment of Lphn2 neuronal distribution likely includes a certain percentage of false negatives/positives of surface associated sites. As such, distributions measured in this study are relative estimates rather than absolute quantifications.

## STAR★Methods

### Key resources table

**Table:** 

REAGENT or RESOURCE	SOURCE	IDENTIFIER
**Antibodies**		

GFP Antibody, Rabbit Polyclonal	Invitrogen	Cat# A-11122; RRID:AB_221569
vGlut1 Antibody, Guinea Pig Polyclonal	Synaptic Systems	Cat# 135–304; RRID:AB_887878
vGlut2 Antibody, Guinea Pig Polyclonal	Synaptic Systems	Cat# 135–404; RRID:AB_887884
Homer1 Antibody, Mouse Monoclonal	Synaptic Systems	Cat# 160–011; RRID:AB_2120992
Goat anti-rabbit Alexa 488 plus	Thermo Fisher Scientific	Cat# A32731; RRID:AB_2633280
Goat anti-mouse Alexa 555 plus	Thermo Fisher Scientific	Cat# A32727; RRID:AB_2633276
Goat anti-guinea pig Alexa 633	Thermo Fisher Scientific	Cat# A21105; RRID:AB_2535757

**Chemicals, peptides, and recombinant proteins**		

Normal Goat Serum	Abcam	ab7481
Streptavidin, Alexa Fluor 555 Conjugate	Thermo Fisher Scientific	S21381
Vectashield Plus Antifade Mounting Medium	Vector Laboratories	H-1900-10
Vectashield Plus Antifade Mounting Medium with DAPI	Vector Laboratories	H-2000-10
Opal 540 Reagent Pack	Akoya Biosciences	FP1494001KT
Opal 620 Reagent Pack	Akoya Biosciences	FP1495001KT

**Critical commercial assays**		

RNAscope Multiplex Fluorescent v2 Reagent Kit	Advanced Cell Diagnostics	323100
RNAscope Probe - Mm-Lphn2	Advanced Cell Diagnostics	319341
RNAscope Probe - Mm-Rbfox3-C2	Advanced Cell Diagnostics	313311

**Experimental models: Organisms/strains**		

Mouse: Lphn2^mVenus^	Anderson et al. 2017	The Jackson Laboratory (JAX: 023401)

**Software and algorithms**		

ImageJ	NIH	https://imagej.nih.gov/ij/
Imaris 9.6+	Oxford Instruments	https://imaris.oxinst.com/
Prism 10	GraphPad Software	https://www.graphpad.com/
MATLAB R2020+	Mathworks Inc.	https://www.mathworks.com/
Imaging ROI Picker (Custom FIJI/ImageJ Macro)	This paper	Zenodo: https://doi.org/10.5281/zenodo.8342580
Close Spots Identification and Bulk Statistics Export (Custom Imaris Extensions)	This paper	Zenodo: https://doi.org/10.5281/zenodo.8368611
Spot Minimum Distances (Custom MATLAB Script)	This paper	Zenodo: https://doi.org/10.5281/zenodo.8368622

**Other**		

High Precision Microscopy slides	Thorlabs	MS10UW
HistoBond+M adhesive slides	VWR	16004–406
Precision glass slide covers	Thorlabs	CG15KH1

### Resource availability

#### Lead contact

Further information and requests for resources and reagents should be directed to and will be fulfilled by the lead contact, Garret R. Anderson (garret.anderson@ucr.edu).

#### Materials availability

This study did not generate new unique reagents.

#### Data and code availability

• All data reported in this study will be shared by the lead contact upon request.
• All original code has been deposited at Zenodo.org and is publicly available as of the date of publication. DOIs are listed in the key resources table.
• Any additional information required to reanalyze the data reported in this paper is available from the lead contact upon request.

### Experimental model and study participant details

#### Animals

Lphn2^mVenus^ transgenic mice used in this study were described previously.^9^ The original mouse line for generation of Lphn2^mVenus^ is available through the Jackson Laboratory Mouse Repository for distribution (B6; 129S6-Adgrl2tm/sud/J, JAX Stock number: 023401). Mice were housed in groups of 2–5 animals at the University of California, Riverside Animal Housing Facility, and were kept on a 12-h light/dark cycle with food and water provided *ad libitum*. Mice were weaned at 21 days of age and were sacrificed between postnatal day 25 and 30 (P25-P30) as described below, with approximately equal numbers of male and female animals used for all experiments. All procedures conformed to National Institutes of Health Guidelines for the Care and Use of Laboratory Mice and were approved by the University of California, Riverside Administrative Panel on Laboratory Animal Care, and Administrative Panel of Biosafety.

### Method details

#### Single molecule RNA fluorescent *in situ* hybridization (single molecule fluorescent in situ hybridization)

*In situ* hybridization for NeuN and Lphn2 was performed as described previously (Donohue et al. 2021). Briefly, P30 wild-type mice were deeply anesthetized with isoflurane and perfused transcardially with ice-cold PBS followed by 4% PFA, both containing 1% diethyl pyrocarbonate (DEPC) to inactivate RNAases. Brains were quickly extracted and postfixed in 4% PFA at 4°C for 24 h, then cryoprotected in stepwise sucrose concentrations, and frozen at −80°C. Frozen brains were cryosectioned at a thickness of 15 μm. *In situ* hybridization was performed using the RNAscope Multiplex Fluorescent v2 assay from Advanced Cell Diagnostics (ACD) according to manufacturer instructions. RNAscope probes for Lphn2 (Mm-Lphn2, 319341; ACD) and Rbfox3 (Mm-Rbfox3-C2; 313311; ACD) were detected using Opal 540 and Opal 620 fluorophores respectively (FP1494001KT and FP1495001KT; Akoya biosciences), and slides were mounted with Vectashield Plus containing DAPI (Vector Laboratories; H-2000-10). SmFISH signals were visualized on a Zeiss LSM 880 confocal microscope with Airyscan using a 10x/0.45 M27 air objective, scanning in airy fast “Flex” resolution mode (0.7x Nyquist) at a speed of 2.17 μs/px. Rbfox3 probe hybridized to Opal 620 was captured using a 561 nm laser with 42 BP0–480 + 49 BP5–620 emission filter and LP 570 secondary beam splitter. Opal 540 (Lphn2) signals were captured using a 488 nm laser with 42 BP0–480 + 49 BP5–550 emission filter and LP 525 secondary beam splitter. Cell nuclei were stained with DAPI and imaged using a 405 nm laser with 42 BP0–480 + 49 BP5–550 emission filter and 42 BP0–460 + LP 500 secondary beam splitter.

Captured ISH images were subsequently analyzed in FIJI/ImageJ. For each slice image, 3 rectangular regions 600 pixels wide (∼132 μm) were drawn radially over the subiculum pyramidal layer, centered on the dSub, iSub and pSub subregions (see Figure 1). Each rectangle was divided into “deep” and “superficial” subregions at the halfway point. Cells in each region were manually counted using the multi-point tool and classified into four groups according to NeuN and Lphn2 expression Each rectangle was divided into “deep” and “superficial” subregions at the halfway point. Cells in each region were counted using the multipoint tool and classified into four groups according to NeuN and Lphn2 expression (NeuN+/Lphn2+, NeuN+/Lphn2-, NeuN-/Lphn2+, and NeuN-/Lphn2-). Lphn2 puncta densities in each group were obtained by manually counting total Lphn2 puncta with the multi-point tool and dividing by total Lphn2+ cells in the group. Puncta counts within dense/overlapping clusters were estimated based on the approximate size of the cluster relative to the average size of isolated puncta.

#### Immunofluorescence

For Lphn2 and synaptic immunofluorescence, Lphn2^mVenus^ transgenic mice were perfused transcardially with 20 mL PBS (137 mM NaCl, 2.7 mM KCl, 10 mM Na_2_HPO_4_, and 1.8 mM KH_2_PO_4_, pH 7.4) and 10 mL fresh 4% paraformaldehyde (PFA) in PBS. Brains were dissected out, placed in 4% PFA at 4°C overnight, briefly rinsed in PBS, and mounted in agarose. Horizontal sections (100 μm) were prepared using a VT1200S vibratome (Leica). Dorsal hippocampal sections were washed for 5 min in PBS and blocked for 1 h at room temperature with 10% normal goat serum (NGS; ab7481, Abcam) and 0.5% Triton X-100 in PBS. Subsequently, slices were incubated overnight at 4°C in PBS containing 1% NGS, 0.01% Triton, and primary antibody. Sections were washed with PBS 3 times for 5 min each and then incubated in PBS containing 1% NGS, 0.01% Triton, and secondary antibody for 4 h at room temperature. Sections were washed again with PBS (3 washes, 5 min each) and then mounted on glass slides (MS10UW, Thorlabs) using Vectashield Plus Antifade mountant (Vector Laboratories, H-1900-10) and #1.5H high-precision cover glass (CG15KH, Thorlabs).

#### Antibodies

Immunofluorescence experiments were performed using the following primary antibodies: anti-GFP for Lphn2^mVenus^ detection (1:1000; rabbit polyclonal; A-11122; Thermo Fisher Scientific), anti-vGlut1 (1:500; guinea pig polyclonal; 135–304; Synaptic Systems), anti-vGlut2 (1:200; guinea pig polyclonal; 135–404; Synaptic Systems), and anti-Homer1 (1:200; mouse monoclonal; 160-011; Synaptic Systems). Primary antibodies were detected using the following fluorophore-conjugated polyclonal secondary antibodies: goat anti-rabbit Alexa-Plus 488 (1:1000 A32731; Thermo Fisher Scientific), goat anti-mouse Alexa-Plus 555 (A32727; Thermo Fisher Scientific), and goat anti-guinea pig Alexa 633 (1:1000 A21105; Thermo Fisher Scientific).

#### Electrophysiology

Lphn2^mVenus^ mice (25–29 days old) were deeply anesthetized with isoflurane and decapitated. Acute horizontal hippocampal slices (300 μm) were prepared using a VT1200S vibratome (Leica) in ice-cold cutting solution containing (in mM) 80 NaCl, 2.5 KCl, 1.25 NaH_2_PO_4_, 25 NaHCO_3_, 6 MgCl_2_, 0.5 CaCl_2_, 1.3 Ascorbic acid, 3 myo-inositol, 2 Na-pyruvate, 11 D-glucose, 73 sucrose, and 0.1 Kynurenic Acid. Slices were recovered in cutting solution at 37°C for 45 min and then transferred to artificial cerebrospinal fluid (ACSF) containing (in mM) 126 NaCl, 2.5 KCl, 1.25 NaH_2_PO_4_, 26 NaHCO_3_, 1.3 MgCl_2_, 2 CaCl_2_, and 11 D-glucose. All solutions were bubbled with 95%/5% O_2_/CO_2_ (carbogen). Slices were allowed to equilibrate in ACSF for at least 15 min before use. For the whole-cell patch clamp, slices were transferred to a recording chamber and continuously infused with oxygenated ACSF using a PeriStar Pro peristaltic pump (World Precision Instruments) and heated to 35°C–36°C with a CL-100 temperature controller and SC-20 in-line solution heater (Warner Instruments). Hippocampal CA1 and subiculum pyramidal neurons were visualized under DIC optics on a Nikon Eclipse FN1 upright microscope equipped with Plan 4x/0.10 and NIR APO 40x/0.8 W objectives and accompanying NIS-Elements AR 3.0 software (Nikon). Whole-cell patch pipettes were pulled from borosilicate glass capillaries (TW150-4, World Precision Instruments) on a PC-10 pipette puller (Narishige) with a tip resistance of 3.5–5.0 MΩ when filled with an internal solution containing (in mM): 137 K-gluconate, 4 KCl, 10 HEPES, 4 Mg-ATP, 0.3 Na_2_-GTP, 10 phosphocreatine, 0.4 EGTA, and 5.37 Biocytin, pH 7.3–7.4 with KOH. Recordings were acquired using a Multiclamp 700B amplifier and Digidata 1550B digitizer, controlled through Multiclamp 700B commander 2.1 and pClamp 10.6 software (Molecular Devices). Neurons were current clamped at 0 pA to record the AP profile and quiescent postsynaptic potential (PSP) activity, collected using a standard current step protocol (−100 to 400 pA in 10 pA intervals) and 2-min gap-free recording, respectively, and sampled at 10 kHz with a 2 kHz Bessel filter. After PSP recording was complete, neurons were held in voltage clamp mode (−70 mV) for an additional 2-5 min to ensure effective biocytin filling, after which the patch pipette was slowly and carefully withdrawn. Successful formation of an outside-out patch (indicated by a smooth, stable return to a GΩ-resistance seal) was considered an indication of cell viability.

Following a successful outside-out seal, slices were allowed to rest for at least 5 min (to ensure full cell recovery and maximal intracellular biocytin spread) before being transferred to 4% paraformaldehyde (PFA). Slices were incubated in PFA with gentle rocking at room temperature for 2 h (or 4°C overnight) before transferring to phosphate-buffered saline (PBS). Biocytin and Lphn2 immunolabelling was performed on the fixed slices as described for immunofluorescence, but with some modifications to improve antibody penetration in the thicker tissue, including: (1) Increasing blocking time to 2 h; (2) Incubating in secondary antibodies at 4°C overnight. Biocytin-filled neurons were visualized at this step with the addition of 2 μg/ml streptavidin Alexa Fluor 555 conjugate (S21381; Thermo Fisher Scientific); and (3) Tissue was washed with PBS at least 4 times for at least 10 min each after the antibody steps.

#### Zeiss airyscan superresolution imaging

Superresolution imaging was performed on a Zeiss LSM 880 confocal microscope equipped with a 32-channel Airyscan detector.^42^ For Lphn2 dendrite localization (post-electrophysiology), Alexa 555 (biocytin, for dendrite morphology) was imaged using a 561 nm laser and Airyscan emission filter sets (495–550 nm bandpass +570 nm long-pass, or 570–620 nm bandpass +645 nm long-pass), along with a 615 nm short-pass filter. Lphn2 puncta were captured using a 488 nm laser and 420–480 nm + 495–550 nm bandpass emission filter set. The laser power, PMT gain, and digital gain for each channel were adjusted as necessary to minimize noise and photobleaching. To determine Lphn2 localization, a 10x or 20x air lens (Plan-Apochromat 10x/0.45, Plan-Apochromat 20x/0.8, or Fluor 20x/0.75, Zeiss) was first used to capture an overview image stack, encompassing the entire X-Y extent of the dendritic tree and spanning ∼100–150 μm deep in the tissue (0.44–1 μm per z section, scanned at 0.52–1.87 μs/px). From this overview, a set of imaging regions was manually selected for high-resolution imaging using a custom FIJI/ImageJ macro (“Imaging ROI picker”) to ensure that the selected imaging regions were within the usable working distance of the objective (typically ∼80–90 μm below the slice surface) and covered the functional domains of the dendritic tree without overlapping. Cells in which any dendrite compartments could not be imaged were excluded from further study. High-resolution image stacks were captured with a Plan-Apochromat 63x/1.4 or 40x/1.4 oil objective (Zeiss) at 2-4x or 6.3x zoom, respectively, scanned at 0.66–2.65 μs/px in “SR” resolution mode (X/Y = 0.043 μm/px; Z = 0.185 μm/px). Refractive index correction factors were applied accordingly (1.45 for air, 0.96 for oil) prior to all z stack captures to minimize z-elongation. Captured image stacks were subsequently processed using the airyscan processing tool in Zen black software (Zeiss) with auto-calculated parameters.

#### 3-Dimensional imaging analysis

Lphn2 dendrite localization was analyzed using Imaris 9.6 3D image analysis software. Each high-magnification image stack was loaded into Imaris and trimmed (if necessary) to remove edge artifacts or black space. Image stacks with a Z-dimension >30 μm were trimmed to <30 μm thick and/or split into separate “sub”-stacks of <30 μm each to limit intensity differences between the top and bottom slices of a stack. After trimming was complete, the Lphn2 channel for each stack was processed with the “subtract background” (1 μm radius) and “Normalize layers” tools to correct for any remaining intensity attenuation along the z axis. Biocytin channels (561 nm) generally required no image preprocessing.

Dendrites and spines were reconstructed using the “Filament” tracing tool. Dendrites were traced semiautomatically using the “autopath” tool, and diameters were calculated automatically by the filament creation wizard followed (when necessary) by manual adjustments. Spines were generated either with the autopath tool or through the creation wizard’s spine detection step (with seed points placed manually), and diameters were adjusted automatically through the creation wizard. Dendrites were split into separate filament objects according to their domain/subdomain classification (see Figure 2), using the full-cell overview stack as a reference. Lphn2 puncta were reconstructed by using the “Spots” creation wizard to detect puncta 0.3 × 0.3 × 0.6 μm in diameter, with the “Different spot sizes” option enabled, and auto-calculated thresholds for spot detection and size. To ensure consistent threshold calculations (which could be biased by puncta density and/or intensity gradients in thicker volumes), threshold parameters were calculated based on the top 10 μm of the stack and used for spot detection over the entire stack. Dendrite-associated spots were identified based on a threshold distance of 0.5 μm from the dendrite, and were categorized as either “shaft-associated” or “spine-associated” depending on which compartment (dendrite shaft or spine respectively) was closest. Custom Imaris extensions (“Close Spots Identification and Bulk Statistics Export”) were used to identify, categorize, and export statistics for all dendrite-associated spots.

Lphn2 puncta counts and dendrite length/area/volume measures were binned to obtain a single puncta density measurement per cell for each of the domain/subdomain categories, which were then averaged across cells for each cell type. For normalized values, Lphn2 density measures for each domain/subdomain category in a given cell were divided by the average density across all categories in the same cell and then averaged by cell type as described above.

For synaptic analysis, the Imaris “Spots” tool was used to detect immunofluorescence puncta (vGlut1, vGlut2, Homer-1, Lphn2) and export their center point coordinates. A custom MATLAB script (“Spot Minimum Distances”) was then used to calculate three-dimensional distances and determine the minimum distance between puncta. Three-dimensional distances between puncta were calculated according to distance formula: d = $\sqrt{{\left(x2-x1\right)}^{2}+{\;\left(y2-y1\right)}^{2}+{\left(z2-z1\right)}^{2}}$. Synaptic overlap analysis was performed using Imaris “Colocalization Volume Statistics” tool. Thresholds for each channel were determined independently by visualization across multiple z-plane sections to minimize non-puncta detection.

### Quantification and statistical analysis

All statistical analyses were performed using Prism 10 software (GraphPad). Data are shown as the mean ± SEM. Significance testing was performed using either one-way ANOVA with post hoc Tukey’s test for multiple comparisons or Mann-Whitney test, as appropriate. Statistically significant differences are indicated by asterisks (∗p < 0.05; ∗∗p < 0.01; ∗∗∗p < 0.001). All relevant data presented in this study are available from the authors.

## Supplemental information

Documents S1. Figures S1 and S2
