# Supplementary material for: Synaptic cell-adhesion molecule latrophilin-2 is differentially directed to dendritic domains of hippocampal neurons
Source: iScience. 2024 Jan 16;27(2):108799. doi: 10.1016/j.isci.2024.108799 (PMC10839266; doi:10.1016/j.isci.2024.108799)
Supplement: Documents S1. Figures S1 and S2 [file mmc1.pdf]

iScience, Volume *27*

Supplemental information

Synaptic cell-adhesion molecule latrophilin-2 is differentially directed to dendritic domains of hippocampal neurons

Thomas R. Murphy, Ryan F. Amidon, Jordan D. Donohue, Libo Li, and Garret R. Anderson

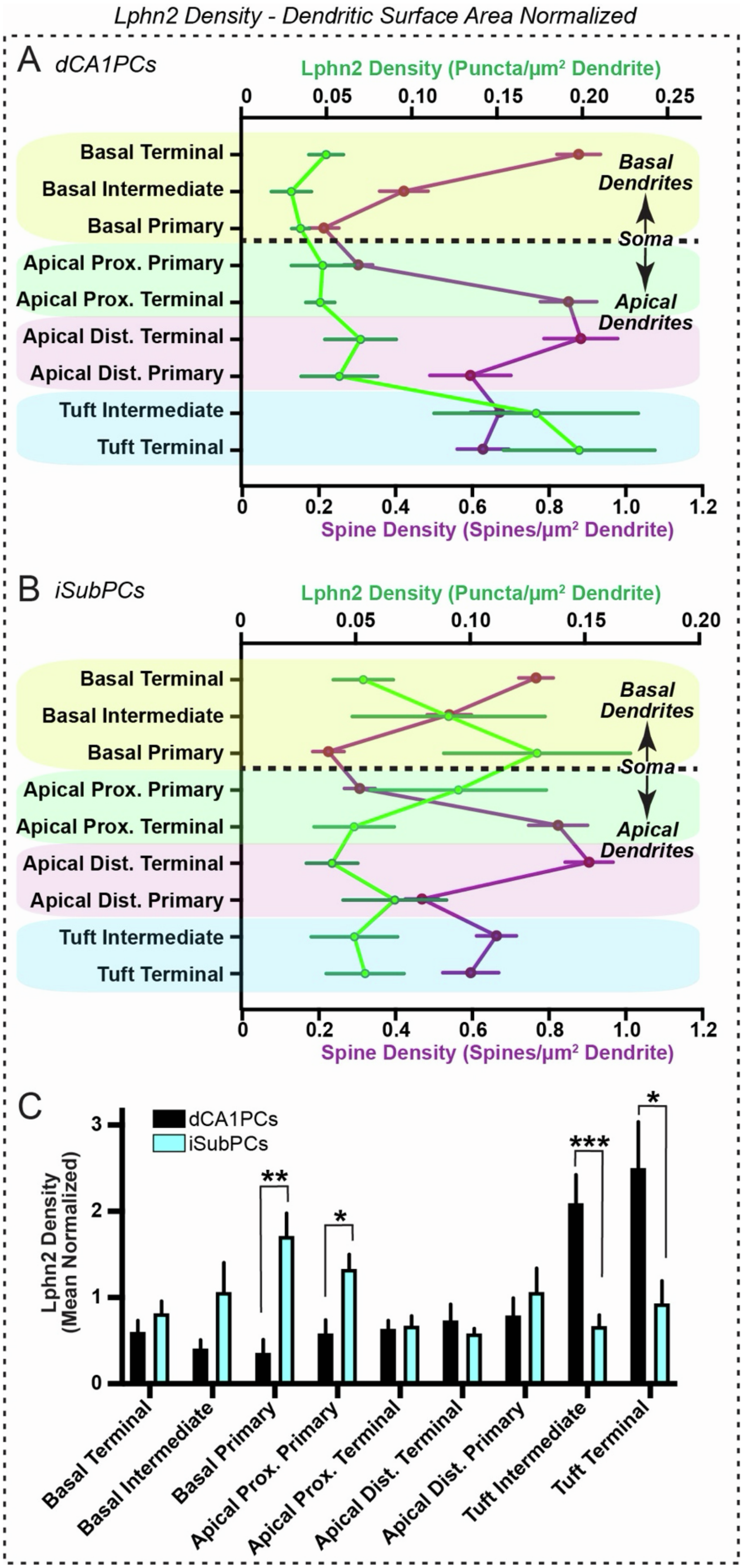

Figure S1. Lphn2 surface density on late-bursting dCA1PCs and early-bursting iSubPCs, Related to Figures 3-4. (**A-B**) Average Lphn2 puncta (green) and spine densities (purple) quantified for each domain normalized by dendritic surface area for late-bursting dCA1PCs (**A**) and early-bursting iSubPCs (**B**). (**C**) dCA1PCs and iSubPC dendritic domain Lphn2 density comparisons (normalized by total Lphn2 density across all dendritic domains). Data are displayed as means ± SEMs (dCA1PCs n=7, iSubPCs n=8 neurons). Statistical analysis was performed by Mann-Whitney test (*p<0.05, **p<0.01, ***p<0.001).

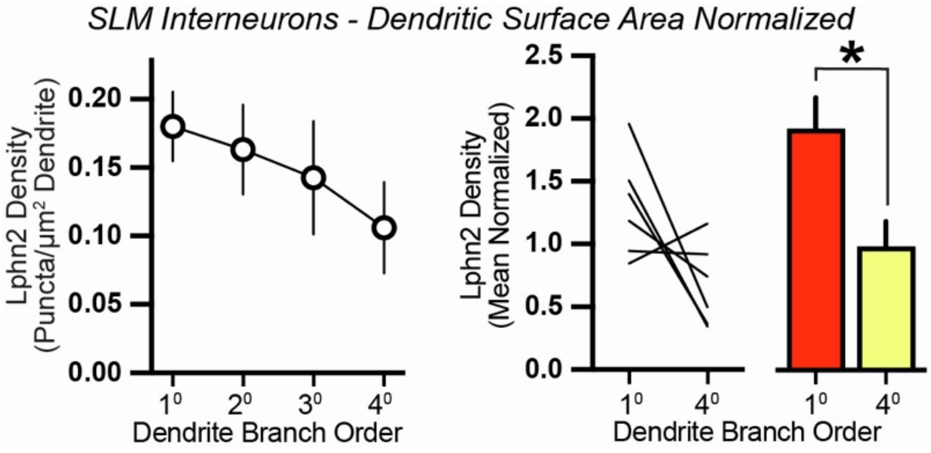

Figure S2. Lphn2 surface density on SLM interneurons, Related to Figure 6. *Left,* Lphn2 contact point density quantifications normalized by dendritic surface area for primary (1^0), secondary (2^0), tertiary (3^0), and quaternary (4^0) dendritic branches of SLM interneurons. *Right,* Single-cell (*left*) and summary graphs (*right*) of Lphn2 densities for 1^0 and 4^0 branch orders, normalized by total Lphn2 density across all dendrites. The data shown are the means +/- SEMs (n = 6 neurons). Statistical analysis was performed by Mann-Whitney test (*$p<0.05$).
